# Supplementary material for: Developing artificial intelligence tools for institutional review board pre-review: A pilot study on ChatGPT’s accuracy and reproducibility
Source: PLOS Digit Health. 2025 Jun 30;4(6):e0000695. doi: 10.1371/journal.pdig.0000695 (PMC12208443; doi:10.1371/journal.pdig.0000695)
Supplement: S11 File — This is a publicly available mock IRB evaluation form published by the Ministry of Health, Labour and Welfare of Japan. It was referenced in determining the provisional KPIs used in this study. Please note that the correctness of the evaluation results in this form has not been publicly verified, and thus cannot be directly compared with the AI-generated outputs. (PDF) [file pdig.0000695.s011.pdf]

整理番号	

西暦２０２０年　２月　５日

技術専門員評価書

認定臨床研究審査委員会
委員長　殿

技術専門員
（所属）
（氏名）

下記の臨床研究について以下のとおり評価しました。

記

実施計画番号*1<br>（jRCT番号）	
研究名称	HER2遺伝子の増幅を認める標準的な治療法のない進行性固形がん患者を対象としたトラスツズマブの第Ⅱ相試験
技術専門員の専門分野	□対象疾患領域（疾患領域名：＿＿＿＿＿＿）<br>□臨床薬理学（□毒性学　□薬力学・薬物動態学　□その他；＿＿＿＿）<br>□生物統計家<br>□医療機器（□臨床工学　□材料工学　□その他；＿＿＿＿）<br>□再生医療<br>■その他；内科学
評価の内容	有効な治療法のない進行性固形がんを対象に、バイオマーカーに基づいた治療選択を試みる研究であり、臨床的な意義は大きいと考えられる。<br>一方で、乳がんや胃がん以外の固形がんでのHER2遺伝子増幅の病態生理学的な意義は十分明らかではなく、特に後発品を治療で使用する本研究においては、HER2遺伝子増幅を標的とする科学的根拠やエビデンスは十分であるとは言えない。したがって、被験者の適格性の判断は、エキスパートパネルによる十分な検討結果に基づき行うことが望ましいと考える。<br>効果判定においては、標的病変・非標的病変の選択と効果判定基準について、特に測定可能病変を有さない場合の判定方法など、研究計画書に明記されておくべきと考える。さらに、画像診断を含む総合効果の判定は、治療開始後12-15週目等、少なくとも一点では必ず実施するよう規定しておくことを検討されたい。<br>また、将来的な抗HER2薬の適応拡大等に際し、本研究のデータ二次利用の必要性は高いと考えられる。実施計画書や説明文書・同意書に、予めデータ二次利用に関する項目を追加しておくことを検討されたい。

*1：新規審査依頼時は記載不要。
注）本書式は技術専門員が作成し、認定臨床研究審査委員会 委員長に提出する。

整理番号	

西暦2020年1月31日

技術専門員評価書

認定臨床研究審査委員会

委員長　殿

技術専門員
（所属）
（氏名）

下記の臨床研究について以下のとおり評価しました。

記

実施計画番号*1<br>（jRCT番号）	
研究名称	HER2遺伝子の増幅を認める標準的な治療法のない進行性固形がん患者を対象としたトラスツズマブの第Ⅱ相試験
技術専門員の専門分野	☐対象疾患領域（疾患領域名：＿＿＿＿＿＿＿＿＿＿＿＿＿＿＿）<br>☐臨床薬理学（☐毒性学　☐薬力学・薬物動態学　☐その他；＿＿＿＿＿）<br>☐生物統計家<br>☐医療機器（☐臨床工学　☐材料工学　☐その他；＿＿＿＿＿＿＿＿）<br>☐再生医療<br>☑☐その他；遺伝医療，ゲノム研究
評価の内容	本申請及び研究計画に特段の問題はないと考えます.<br>審査手続きを進めていただければと思います.

*1：新規審査依頼時は記載不要。

注）本書式は技術専門員が作成し、認定臨床研究審査委員会 委員長に提出する。

参考書式１

整理番号	

西暦　　　年　　月　　日

技術専門員評価書

認定臨床研究審査委員会

殿

技術専門員

下記の臨床研究における科学的観点での評価について以下のとおり報告いたします。

記

実施計画番号*1<br>（jRCT番号）		
研究名称	HER2遺伝子の増幅を認める標準的な治療法のない進行性固形がん患者を対象としたトラスツズマブの第Ⅱ相試験	
技術専門員の<br>専門分野	■　疾患領域の専門家<br>□　臨床薬理学の専門家<br>□　生物統計の専門家<br>□　その他の専門家（　　　　　　　　　　　）	
研究の妥当性	■あり　　□なし	
専門的評価	臨床研究の特徴	
	NGS にて HER2 遺伝子増幅が検出された乳癌、胃癌以外の固形癌を対象とした、抗 HER2 抗体であるトラスツズマブの抗腫瘍効果を評価するバスケット型臨床試験である。	
	注意すべき点	

参考書式１

整理番号	

1,2 研究スケデュール
心機能評価- 登録前28日以内でよろしいのではないでしょうか。

6.1.1患者選択基準
(2) 遺伝子増幅の定義となるコピー数を明記してください。
(3) 奏効率を主要評価項目にした試験ですが、評価可能病変のみの症例も登録可能となっているのはいかがなものでしょう。再考ください。

6.1.2除外基準
(3) トラスツズマブ抵抗性に関わる遺伝子変異は定義できますか？もしできないようであれば削除することを勧めます。
(9) 左室駆出率（LVEF）が 50%未満である患者；50%以上として選択基準にいれることを勧めます

6.2 目標症例数
【設定根拠】第2段階では20例で合計41例でしょうか。ご確認ください。第一段階で何例奏効がでれば第2段階に進むのかをここにもご記載ください。

12.4 臨床検査
(4) HBV-DNA定量はHBs抗原、HBc抗体が陽性の場合もモニタリングが必要です。4週毎にモニタリングとなっていますが、研究スケデュール表では6週毎となっています。ご確認ください。
(6) 心機能評価：EFが途中で低下した場合の中止基準、再開基準を明記ください。

考えられるメリット・デメリット

メリット：分子診断に基づいた治療であり、本来治療のオプションのない患者に有効な治療を届けられる可能性がある。

デメリット：トラスツズマブ単剤での効果は乳癌の前例から推測しても限定的であり、より新しい抗HER2薬に比べると魅力に欠ける。

総評

HER2遺伝子増幅は遺伝子パネル検査で比較的高頻度に検出される遺伝子変化であり、本バスケット試験で抗腫瘍効果が認められれば、tumor agnosticなトラスツズマブの開発に結びつく可能性があり意義深いと考えます。

*1：新規審査依頼時は記載不要。
注）本書式は技術専門員が作成し、認定臨床研究審査委員会に提出する。

参考書式１

整理番号	

西暦 2020年　01月　14日

技術専門員評価書

認定臨床研究審査委員会

委員長　殿

技術専門員
（研究機関名）
（所属・職名）
（氏　　　名）

下記の臨床研究について以下のとおり評価しました。

記

実施計画番号*1<br>（jRCT番号）	
研究名称	HER2遺伝子の増幅を認める標準的な治療法のない進行性固形がん患者を対象としたトラスツズマブの第Ⅱ相試験
技術専門員の専門分野	■対象疾患領域<br>（疾患領域名：HER2遺伝子の増幅を認める標準的な治療法のない固形がん）<br>□臨床薬理学（□毒性学　□薬力学・薬物動態学　□その他；＿＿＿＿＿）<br>□生物統計家<br>□医療機器（□臨床工学　□材料工学　□その他；＿＿＿＿＿）<br>□再生医療<br>□その他；＿＿＿＿＿
評価の内容	本内容は標準的な抗がん治療の効果を期待できないか、標準的な抗がん治療のない固形がん患者に対し、保険診療の「がん遺伝子パネル検査」により HER2 増幅が認められた場合にトラスツヅマブ投与を行う第Ⅱ相試験である。<br>今後、がん医療の個別化が求められていく中で、このようなバスケット型臨床試験の実施は不可欠であり、内容的にも妥当なものとなっている。<br>以下に質問事項を上げる。<br>① 標準的な抗がん治療の解釈が難しい。本邦の臓器別ガイドラインを準拠し判断するのか、NCCN ガイドラインや ESMO ガイドライン等も含めるのか。とくに、稀少がんや原発不明がん、小児がんではどのように判断するのか、その領域の専門家の判断も仰ぐのか等、具体的な説明が望まれる<br>② トラスツヅマブ先発品ではなく後発品を用いる内容であるが、研究タイトルからは誤解を受けないか、具体的な薬品名も必要ではないか。

*1：新規審査依頼時は記載不要。
注）本書式は技術専門員が作成し、認定臨床研究審査委員会 委員長に提出する。

※模擬審査

参考書式１

整理番号	

西暦　2020　年　1月　13日

技術専門員評価書

認定臨床研究審査委員会
委員長　殿

技術専門員
（所属）
（氏名）

下記の臨床研究について以下のとおり評価しました。

記

実施計画番号[*1]（jRCT番号）	HER2 遺伝子の増幅を認める標準的な治療法のない進行性固形がん患者を対象としたトラスツズマブの第Ⅱ相試験
研究名称	HER2遺伝子の増幅を認める標準的な治療法のない進行性固形がん患者を対象としたトラスツズマブの第Ⅱ相試験
技術専門員の専門分野	■対象疾患領域（疾患領域名：　消化器内科学・　腫瘍内科学　）<br>□臨床薬理学（□毒性学　□薬力学・薬物動態学　□その他；＿＿＿＿＿）<br>□生物統計家<br>□医療機器（□臨床工学　□材料工学　□その他；＿＿＿＿＿＿＿＿）<br>□再生医療<br>□その他；＿＿＿＿＿＿＿＿＿＿＿＿＿＿＿＿＿＿＿＿
評価の内容	**研究の意義**：現在、HER2 遺伝子増幅を高頻度に認める乳がんと胃がんに対して、抗 HER 抗体薬トラスツマブの使用が承認されている。しかし、実際には、胆管がん、尿路上皮がんなどの他の固形がんでも数%に HER2 遺伝子増幅を認める。2019 年にがん遺伝子パネル検査が保険収載されたことから、HER2 遺伝子増幅陽性の固形がんが見出される可能性が高まっている。遺伝子パネル検査は、標準的治療法が無い患者を対象が適応であり、そうした患者を対象にトラスツズマブ BS の有効性を調べる臨床データを集積することは大変有意義である。<br>**安全性について**：トラスツズマブは比較的副作用の少なく、臨床試験としては比較的実施しやすい薬剤である。トラスツズマブは心毒性を有することが報告されているが、登録前にそれらに対する検査も実施することになっており、概ね被験者の安全性は保たれていると考える。ただし、抗体薬であるトラスツズマブ BS には低頻度ながらアナフィラキシーショックがあると考えられ、他にも間質性肺炎、ニューロパチーなども報告されているので、これらのことを同意説明文書にしっかりと記載するべきである。また、計画書の中にも、これらの副作用に十分留意する旨を記載するべきである。<br>**倫理性について**：計画書には、トラスツズマブ BS の治療費に関する記載が無いが、このことがもっとも重要である。通常、1ヶ月に 10 万円ぐらいの治療費がかかると推定されるので、会社負担が望ましいと考える。これらのことを同意説明文書にも明記する必要性がある。ま

	た、計画書には、本治療により健康被害が発生した場合、通常の保険診療で治療すると書かれている。本研究のような臨床試験により副作用が出現して治療を行う場合、保険診療で対応できるのかどうかも確認する必要がある。<br>**その他**：除外基準(3)に「トラスツズマブに対する抵抗性変異」と記載されているが、具体的にどのような変異なのか記載するべきである。また、本試験には大腸がんも含まれているが、HER2 遺伝子増幅大腸がんで既にいろいろな臨床試験が行われている。そのため、大腸がんを含めるのであれば、その根拠を説明する必要がある。<br>**総評**：本臨床試験は、標準的治療法の無い HEER2 遺伝子増幅固形がんを対象にトラスツズマブ BS の有効性を調べる大変有用な臨床試験である。安全性については、概ね問題無いと考えるが、上記のごとく同意説明文書に治療費、同意書における副作用の説明、等を明記する必要がある。また、副作用が出現した場合の対応が保険診療内でカバーできるのかどうか確認するべきである。

*1：新規審査依頼時は記載不要。
注）本書式は技術専門員が作成し、認定臨床研究審査委員会 委員長に提出する。

書式1　　　　　　　　　　　　　　　　　　　　　　　　　　　2020年　1月　21日

技術専門員評価書

殿

技術専門員　所属：[redacted]

役職・氏名：[redacted]

下記の臨床研究における科学的観点での評価について以下のとおり報告いたします。

記

臨床研究実施計画番号*	
臨床研究課題名	HER2遺伝子の増幅を認める標準的な治療法のない進行性固形がん患者を対象としたトラスツズマブの第Ⅱ相試験
技術専門員の専門分野	臨床薬理学
研究の妥当性	■あり　　□なし
専門的評価	臨床研究の特徴
	治療法のない固形がん患者を対象に、「Foundation One CDx がんゲノムプロファイル」等の検査システムで HER2 遺伝子の増幅が認められた患者に、マッチングした治療法の一つであるトラスツズマブ BS を用いて有効性の検討を行う探索的研究である。
	注意すべき点
	①本研究では、SCRUM-Japan、MASTER KEY プロジェクトおよび AMED の革新的がん医療実用化研究事業の中で抗 HER2 療法の医師主導臨床試験や臨床研究との差異が不明である。今回、本試験をやる意義を記載するべきである。<br>②HER2 過剰発現以外の遺伝子異常が同定され他の治療法も推奨される場合、本研究への参加はどのように判断するのかについて記載するべきである。<br>③2施設、2年間で41例を集めるのは、疾患の性質上やや困難と思われるが、実施可能か。<br>④目標症例数の設定根拠で、乳がん以外の患者のトラスツズマブの奏効率があれば、そちらを参考に期待奏効率、閾値奏効率を設定した方がよいと考えられる。
	考えられるメリット・デメリット
	（メリット）<br>①治療法のない固形がん患者を対象に、HER2 遺伝子の増幅が認められた患者への新たな治療法が示される可能性がある。<br>②既に製造販売承認をされているバイオシミラーであり、安全性については十分な情報が得られている。また添付文書の用法用量に基づく使用方法であり、薬剤のリスクは一般臨床のそれを上回るものではない<br>（デメリット）<br>出口戦略が不明であるため、ただ症例集積をし、データ収集のみで、次のステップにつながることなく研究が終了する可能性がある。
	総評
	治療法のない固形がん患者を対象に、HER2 遺伝子の増幅が認められた患者への新たな治療法が示される可能性がある一方で、もし薬事承認を目指しているのであれば、規制要件を再考し、医師主導治験を行うことを推奨する。

注）本書式は技術専門員が作成し、臨床研究審査委員会に提出する。

書式1　　　　　　　　　　　　　　　　　　　　　　　　　　　　　　　　2020年1月21日

技術専門員評価書

殿

技術専門員　所属：[redacted]

役職・氏名：[redacted]

下記の臨床研究における科学的観点での評価について以下のとおり報告いたします。

記

臨床研究実施計画番号*		
臨床研究課題名	HER2遺伝子の増幅を認める標準的な治療法のない進行性固形がん患者を対象としたトラスツズマブの第Ⅱ相試験	
技術専門員の専門分野	呼吸器腫瘍内科	
研究の妥当性	□あり　　■なし	
専門的評価	臨床研究の特徴	
	がん遺伝子パネル検査が保険収載され、治療標的となる遺伝子異常を検査することが可能となったが、判明した遺伝子異常に対して、アクセス可能なマッチする治療は限定的であることが問題である。本研究では、比較的頻度が多い遺伝子異常で、また乳がん・胃がんでの臨床データも豊富なHER2増幅に着目し、がん遺伝子医療のアンメットニーズの解決を目指すという点で臨床的意義があるものと思われる。	
	注意すべき点	
	1)　遺伝子変異と異なりHER2増幅に関しては、いずれの値を持ってcutoffとするかが一般的に曖昧であり、患者選択基準にはその点の記載が認められない。<br>2)　除外基準(3)にある、「トラスツヅマブ抵抗性変異として既知のがん遺伝子」も具体的な遺伝子の記載が無く曖昧である。<br>3)　症例設定の根拠として、HER2陽性乳がんの一次治療としてのトラスツヅマブの奏功率26%を参考としているが、乳がんに関してはIHC法またはFISH法で確認されたHER2強陽性例であり、(上記のHER2遺伝子増幅のcutoff値にもよると思われるが) HER2遺伝子増幅かつ既治療症例においてこれだけの奏功率を期待できるかどうかは不明である。その点で、Simonの二段階法を採用している点は妥当と思われるが、下限閾値の5%が、果たして臨床的意義のある有効性として認められるかどうかも疑問が残る。<br>4)　また、最大の問題点は、そもそも、既に幾つかのトラスツズマブと抗がん剤の抗体薬物複合体が、HER2陽性固形がんに対して一定の有効性を示している中で(e.g. Ann Oncol 11;1821, 2019)、薬効の劣るトラスツズマブ単独治療の有効性を探索することである。	
	考えられるメリット・デメリット	
	メリット：有望な治療の乏しいHER2陽性がん患者に対して有効な治療選択肢となりうる。トラスツズマブ単剤は比較的毒性も低いため患者QoLという点でも通常の抗がん剤よりも良好であると期待できる。<br>デメリット：トラスツズマブ単剤の有効性はあまり高くないと思われるため、本研究への参加が、(少ないと思われるが) 有害事象のリスクや、その他の有効な治療機会の喪失などを引き起こす可能性がある。	
	総評	
	目的やデザインは概ね妥当と思われるが、複数のトラスツズマブの抗体薬物複合体がHER2陽性固形がんでの臨床効果を証明している現状では、薬効の劣るトラスツズマブ単剤を用いて本研究を行う意義は乏しいと考える。<br>また、研究計画書の除外基準4)-6)に関して重複した文章が並んでいるため、整理し書き直す必要がある。	

注）本書式は技術専門員が作成し、臨床研究審査委員会に提出する。
