# Supplementary material for: Developing artificial intelligence tools for institutional review board pre-review: A pilot study on ChatGPT’s accuracy and reproducibility
Source: PLOS Digit Health. 2025 Jun 30;4(6):e0000695. doi: 10.1371/journal.pdig.0000695 (PMC12208443; doi:10.1371/journal.pdig.0000695)
Supplement: Attachment — Submitted filename: Response_to_Reviewers.docx [file pdig.0000695.s013.docx]

**Responses to Reviewers**

We sincerely thank the reviewers for their thoughtful and constructive comments. These insights have greatly contributed to improving the clarity, rigor, and overall quality of our manuscript.

Before responding to each individual comment, we would like to clarify the purpose and scope of our study.

This study is part of a foundational project aimed at developing AI tools to assist Institutional Review Board (IRB) *pre-reviews*. Importantly, the project does not seek to automate or replace ethical decision-making by IRBs. Rather, the goal is to support IRB reviewers by developing explainable AI tools that can assist with document evaluation.

As a pilot study, the primary aim of this research was to assess whether generative AI can accurately and consistently extract and summarize critical information—such as the research objectives, study design, and potential benefits/risks—from Japanese clinical research protocols and informed consent forms (ICFs).
At this early stage, we have not yet evaluated whether the extracted content aligns with IRB-specific review criteria. Instead, we focused on evaluating the accuracy and reproducibility of information extraction and summarization as a baseline capability.

For benchmarking, we adopted a manual evaluation approach, comparing GPT outputs with the original protocol content through visual inspection by a single researcher. This served as a preliminary method in preparation for the next phase, which will involve the implementation of retrieval-augmented generation (RAG). In the next phase, we will assess whether AI can appropriately reference and apply IRB review standards.

**Future Research Plan:**

**Phase 1**: *Baseline evaluation of GPT performance* (current pilot study)

- Assess GPT accuracy and reproducibility in extracting IRB-relevant elements

**Phase 2**: *Introduction of RAG for alignment with IRB criteria*

- Evaluate whether AI can reference IRB review standards to assess the appropriateness of extracted content

**Phase 3**: *Development and validation of multidimensional evaluation methods*

- Establish optimal evaluation metrics through multiple methods and approaches, such as:
  - Agreement with IRB reviewers (e.g., Cohen’s kappa)
  - Comparison with review criteria (to assess alignment with standards)
  - Quantitative NLP-based metrics (e.g., BLEU, ROUGE)
  - Semantic similarity via vector-based approaches (e.g., Sentence-BERT)
  - Exploration of additional statistical approaches

The findings from this pilot study provide essential baseline data for the future integration of RAG and fine-tuning techniques. Ultimately, we aim to develop AI tools that can assist with IRB pre-review tasks by integrating insights from multiple expert perspectives.

To more clearly communicate the focus of our study, we have revised the manuscript title to:
**"Developing artificial intelligence tools for institutional review board pre-review: A pilot study on ChatGPT’s accuracy and reproducibility"**
This change highlights that our study does not involve ethical judgment by AI but instead explores the potential of AI in supporting IRB pre-review processes.

We provide detailed responses to each reviewer comment below. We hope that the manuscript revisions have appropriately addressed all the concerns raised.

**Responses to Reviewer #1**

We sincerely thank Reviewer #1 for the thoughtful and constructive comments, which have greatly contributed to improving the clarity and rigor of our manuscript. Below, we provide detailed responses to each point raised, also clarifying the corresponding revisions made to the manuscript.

**Comment 1: Citation of Godwin et al. (2024)**

*Consider integrating the following reference into your work: Godwin, R. C., et al. (2024). IRB-draft-generator: A generative AI tool to streamline the creation of institutional review board applications. SoftwareX, 25, 101601.*

**Response:**
Thank you for this valuable suggestion. We have incorporated the recommended reference (Godwin et al., 2024) into the Introduction section. Additionally, we clarified the distinction between their work and ours. While Godwin et al. focused on AI tools to support the drafting of IRB applications, our study specifically evaluates whether generative AI can accurately and reproducibly extract and summarize key elements from Japanese-language clinical research protocols and informed consent forms (ICFs). This helps highlight the novelty of our study within the domain of AI-assisted IRB review processes.

**Comment 2: Task-specific fine-tuning and terminology-related errors**

*The study highlights the accuracy of GPT models but does not address the potential for task-specific fine-tuning...*

**Response:**
We appreciate this important observation. As a pilot study, our research focused on evaluating the baseline performance of GPT-4 and GPT-4o using prompt engineering only, without applying any fine-tuning or additional domain-specific datasets. This decision to limit the scope to assessing the foundational capabilities of the models was intentional.
However, we agree that fine-tuning with medical-domain datasets could significantly improve performance, particularly in handling complex or abbreviated medical terminology.
We have now added a statement in the Discussion section indicating that domain-specific fine-tuning is a planned future step, particularly following the integration of retrieval-augmented generation (RAG) to improve the model’s ability to reference IRB review criteria.
We have also added specific examples of terminology-related inaccuracies—such as the omission of "BS" in "trastuzumab BS"—and provided the original Japanese text (Table S1) and GPT-generated outputs (Tables S2 and S3) in the Supporting information section to ensure transparency.

**Comment 3: Limitation regarding in-person site visits**

*One critical limitation is the inability of generative AI to replicate in-person site visits...*

**Response:**
Thank you for highlighting this important point. We have clarified in the Limitations section that our study focuses exclusively on the document-based (written) pre-review process typically used in IRB/CRB reviews in Japan. Since in-person site visits are not commonly part of these domestic procedures, they were not within the scope of this study.

Nonetheless, we have acknowledged this limitation and stated that AI tools cannot replicate human judgment in on-site evaluations. We have added a statement that this will be an important consideration if future applications of AI are expanded to include international IRB practices where in-person site assessments may be standard practice.

**Comment 4: Details on custom GPT development**

*The methodology lacks details on how custom GPTs were developed...*

**Response:**
We agree that additional clarification was necessary. In response, we have expanded the "Materials and methods" section to clearly state that we did not use additional datasets or fine-tuning. The four custom GPTs were developed solely using OpenAI’s GPT customization feature (prompt engineering) without training on new data.

To demonstrate the added value of customization, we included a direct comparison between the outputs of custom GPTs and those of standard prompts using the same research protocol and evaluation metrics (see Tables 4 and 5). This comparison highlights the improved stability and reproducibility achieved through a custom GPT configuration.

**Comment 5: Justification for 10 iterations**

*The manuscript does not clearly explain the basis of 10 iterations...*

**Response:**
Thank you for pointing this out. The choice of 10 iterations was based on practical constraints, given that each evaluation was performed manually. We have now added this rationale to the "Materials and methods" section. In addition, we have clarified in the revised “Limitations section” that this number was pragmatically chosen for this proof-of-concept phase pilot study, and that no statistical validation such as power analysis or convergence testing was performed. This decision aligns with existing guidelines and literature on pilot study design. We agree that further analysis is needed to determine whether the number of runs affects output stability and convergence. As noted in the revised Discussion and Limitations sections, we plan to conduct convergence and sensitivity analyses in future work, including exploring how output variability changes with different numbers of iterations.

**Comment 6: Generalizability across protocols**

*While the study focuses on a single research protocol, the data source reportedly included multiple protocols...*

**Response:**
We appreciate this suggestion. To ensure a controlled analysis in this pilot study, we selected one representative protocol to focus on the core evaluation tasks. However, we agree that broader validation is essential. Therefore, we have indicated in the Discussion section that future studies will evaluate multiple research protocols across diverse disease areas to enhance the generalizability of our findings.

**Comment 7: Lack of benchmark or baseline**

*What benchmark or baseline was used to evaluate the models?...*

**Response:**
Thank you for your question. We acknowledge the absence of a formal benchmark in this pilot study. As we explained in the Limitations section, our current evaluation was based on visual comparison between the AI output and the original Japanese documents using predefined consistency criteria.

That said, we agree that incorporating more rigorous benchmarks will improve the robustness of our evaluation. Accordingly, we have outlined plans in the revised manuscript to use multiple benchmarking approaches in future phases, including:

- Comparison with human IRB reviewer judgments (e.g., Cohen’s kappa)
- Alignment with IRB criteria using RAG
- Quantitative evaluation with BLEU/ROUGE scores
- Similarity scoring with Sentence-BERT

These enhancements are planned as part of Phase 3 in our future research framework.

**Comment 8: Ethical focus in the title**

*While the manuscript addresses AI accuracy and reproducibility, it provides limited discussion on ethics...*

**Response:**
Thank you for this suggestion. We have revised the manuscript title to more accurately reflect the actual focus of the study:

**"Developing artificial intelligence tools for institutional review board pre-review: A pilot study on ChatGPT’s accuracy and reproducibility"**

This change helps clarify that our work does not attempt to replicate ethical reasoning but rather supports IRB members during the document evaluation phase.

**Comment 9: Sample review form**

*Including a sample review form would help clarify the scope and nature of the analysis...*

**Response:**
We have addressed this by adding a mock IRB review form, published by Japan’s Ministry of Health, Labour and Welfare, to the supplementary information. This form includes sample evaluation items and was used as a reference when defining our evaluation categories. However, since the assessment results on that form are not publicly validated, it was used only as a reference, not as a ground truth benchmark.

**Comment 10: Repetitive descriptions of GPT-4o’s performance**

*The authors should avoid repetitive descriptions, particularly regarding GPT-4o’s performance consistency...*

**Response:**
Thank you for this helpful comment. We have reviewed the manuscript and revised both the Results and Discussion sections to remove redundancy and consolidate the description of GPT-4o’s performance. The new structure presents the findings more concisely, improving overall readability while retaining the key information.

**Responses to Reviewer #2**

We sincerely appreciate your thoughtful and constructive comments. Your feedback has significantly contributed to improving the clarity, rigor, and completeness of our manuscript. Please find our detailed responses to each point below. We hope that the revisions have successfully addressed the concerns you raised.

**1. Data Availability**
**Reviewer Comment:** The manuscript does not fully satisfy PLOS Digital Health’s data availability requirements.
**Response:**
Thank you for pointing this out. To clarify, we have provided the Data Availability Statement in the designated field of the PLOS submission system, as required by *PLOS Digital Health*. This field is separate from the manuscript file itself, and the statement will be published as part of the final article upon acceptance.

Specifically, the extracted and structured data used in this study have been deposited in the Figshare repository under the DOI:

[10.6084/m9.figshare.28838291]

[10.6084/m9.figshare.28737845].

We have also prepared a comprehensive set of Supporting Information files (S1–S11), including the original Japanese source texts, English translations, GPT outputs (from both custom and standard prompts), and the mock IRB review form used in our evaluation. These files are provided to enhance transparency and reproducibility in accordance with the PLOS data sharing policy.

The captions of all Supporting Information files are listed at the end of the manuscript, ensuring better alignment between the manuscript and the supplementary materials and facilitating reproducibility.

**2. Methodological Rigor**
**Reviewer Comment:** Important details such as inter-rater reliability or confidence intervals are missing, and the criteria for defining accuracy and reproducibility are not well-articulated.
**Response:**
We acknowledge that our evaluation relied on qualitative manual assessment due to the pilot nature of this study. To clarify, the study focused on exploring ChatGPT’s baseline ability to extract and summarize key elements from research protocols and ICFs without fine-tuning. We conducted 10 iterations per task to compare GPT-4 and GPT-4o, as well as custom GPTs versus standard prompts.

To address your comment, we have made the following revisions:

1. Expanded the Materials and methods section to describe the criteria for accuracy and reproducibility in more detail
2. Clarified in the Limitations section that future studies will introduce statistical validation metrics, such as Cohen’s kappa and confidence intervals, to enhance methodological rigor

**3. Reproducibility**
**Reviewer Comment:** The study’s reproducibility is limited by insufficient detail on datasets and evaluation metrics.
**Response:**
Thank you for pointing this out. In the revised Materials and methods section, we now clearly describe the data source—mock protocols and ICFs from Japan’s Ministry of Health, Labor and Welfare. These materials are publicly available and were used in IRB training contexts.

We also elaborated on the evaluation framework using pre-defined criteria (Tables 7–9) to categorize outputs as “Consistent,” “Partially Consistent,” or “Inconsistent.” All evaluations were conducted based on visual inspection by one reviewer, as clarified in the text.

Additionally, we describe the GPT customization process using OpenAI’s “GPTs” feature (prompt engineering only), without any dataset-based fine-tuning. While the full prompts are not publicly disclosed, we outlined their structure and scope in Table 6.

To enhance reproducibility in future work, we plan to:

- Introduce multiple evaluators and assess inter-rater agreement using Cohen’s kappa
- Apply quantitative NLP metrics such as BLEU, ROUGE, and Sentence-BERT for content similarity
- Use the API for automatic logging and traceability

**4. Ethical Considerations**
**Reviewer Comment:** Ethical risks such as hallucination and bias in generative AI are not sufficiently addressed.
**Response:**
Thank you for your comment. We have addressed the issues of biases and hallucinations in AI-generated outputs in the Introduction section of the manuscript, highlighting that these pose ethical risks. We also emphasize that transparency and reliability are crucial in AI outputs, and Explainable AI (XAI) plays a key role in mitigating such risks.

This project aims to use AI as a supportive tool in the IRB review process, not for the full automation of decisions. AI-generated outputs assist the decision-making process, but the authority to make the final decision must remain with human reviewers. This helps reduce the risk of biases or hallucinations directly impacting the review outcomes.

The study focuses on evaluating whether AI can accurately extract key information such as the research objectives and background, research design, and advantages and disadvantages in the IRB review process. It does not address decision-making based on established criteria, which will be addressed in future work using RAG and fine-tuning (FT) to provide AI outputs that are both interpretable and aligned with review standards. These efforts will help reduce biases and hallucinations and further develop ethically sound AI tools.

**5. KPIs and Performance Metrics**
**Reviewer Comment:** The presented KPIs are preliminary and lack justification.
**Response:**
Thank you for this helpful comment. We now explicitly state in the **Materials and methods** and **Limitations** sections that the KPIs used—accuracy and reproducibility—are **tentative and exploratory** in nature.

We also outline a future roadmap to refine these KPIs using the following approaches:

- Comparing AI outputs with those of multiple human IRB reviewers and calculating **Cohen’s kappa**
- Applying NLP-based metrics (**BLEU**, **ROUGE**, **Sentence-BERT**)
- Comparing outputs against established IRB review criteria
- Introducing a hybrid human-AI assessment pipeline

**6. Figures and Tables**
**Reviewer Comment:** Figure and table captions are not sufficiently detailed.
**Response:**
We have revised all the figure and table captions to ensure that they are **self-contained** and comprehensible without excessive reference to the main text. Each caption now clearly explains the table/figure content, context, and interpretation to improve reader understanding.

**7. Writing and Presentation**
**Reviewer Comment:** Redundancy and overly technical language limit accessibility, particularly in the Abstract and Discussion.
**Response:**
We appreciate your evaluation. We carefully revised the **Abstract** and **Discussion** sections to:

- Eliminate repetitive expressions
- Simplify overly technical terminology
- Improve overall readability while retaining academic precision

These changes were made to ensure broader accessibility and clearer communication of our study’s significance.

We thank you once again for your insightful and constructive feedback. We believe the revised manuscript has greatly improved thanks to your feedback and hope that it now meets the publication standards of *PLOS Digital Health*.
